# Exploring the dynamic interplay between learning and working memory within various cognitive contexts

**DOI:** 10.3389/fnbeh.2024.1304378

**Published:** 2024-02-14

**Authors:** Zakieh Hassanzadeh, Fariba Bahrami, Fariborz Dortaj

**Affiliations:** ^1^Faculty of Psychology and Educational Sciences, Allameh Tabataba’i University, Tehran, Iran; ^2^School of Electrical and Computer Engineering College of Engineering, University of Tehran, Tehran, Iran

**Keywords:** reinforcement learning, working memory, MoCA, RLWM model, aging

## Abstract

**Introduction:**

The intertwined relationship between reinforcement learning and working memory in the brain is a complex subject, widely studied across various domains in neuroscience. Research efforts have focused on identifying the specific brain areas responsible for these functions, understanding their contributions in accomplishing the related tasks, and exploring their adaptability under conditions such as cognitive impairment or aging.

**Methods:**

Numerous models have been introduced to formulate either these two subsystems of reinforcement learning and working memory separately or their combination and relationship in executing cognitive tasks. This study adopts the RLWM model as a computational framework to analyze the behavioral parameters of subjects with varying cognitive abilities due to age or cognitive status. A related RLWM task is employed to assess a group of subjects across different age groups and cognitive abilities, as measured by the Montreal Cognitive Assessment tool (MoCA).

**Results:**

Analysis reveals a decline in overall performance accuracy and speed with differing age groups (young vs. middle-aged). Significant differences are observed in model parameters such as learning rate, WM decay, and decision noise. Furthermore, among the middle-aged group, distinctions emerge between subjects categorized as normal vs. MCI based on MoCA scores, notably in speed, performance accuracy, and decision noise.

## 1 Introduction

What exactly is memory and is it connected to the learning or are they distinct concepts? In some languages such as Farsi, they share a common linguistic root which suggesting a familial connection between the two. However, in terms of their functions within the brain, we find two distinct yet complementary systems at play: the dopaminergic corticostriatal circuitry associated with reinforcement learning (RL) and the circuitry responsible for working memory (WM), which is a part of the prefrontal cortical executive function system. The concept of associative or instrumental learning has always been tied to the concept of memory. The learning happens as soon as the agent is able to remember or recall the stimulus-action pair. The pair which is being reinforced in some steps before by means of reward signals such as dopamine. Here, delay is a key factor. If there are some steps or a time interval between the stimulus and the desired response, the mechanism through which learning is happening is called reinforcement learning. If the interval is very short to be called immediate, the associative learning lies in the category of working memory. This is the case because WM is capable of the immediate and exact storage and manipulation of data with decay in time (Collins and Frank, 2012; Collins et al., 2014; Gold et al., 2017; van de Vijver and Ligneul, 2019), while RL works slowly and robust in long term and is representative of sequential problems in machine learning (Yoo and Collins, 2021). The latter shapes the associations between stimulus-action by the help of a signal representing reward prediction error (RPE), while WM is affected by the delay and load in the related tasks (Collins and Frank, 2012). Studies have shown that brain’s implementation of RL involves interacting with other processes in prefrontal and parietal cortices.

Recent research supports the hippocampus’s critical role in high-precision bindings over brief delays, challenging traditional views and contributing to the ongoing discourse on the hippocampus’s involvement in working memory processes (Yonelinas, 2013; Rubin et al., 2014; Moscovitch et al., 2016; Borders et al., 2022). However, the Reinforcement Learning Working Memory (RLWM) model (Collins and Frank, 2012) guides our investigation, treating the working memory subsystem as an immediate learner with a learning rate (alpha) of 1. This approach enables a focused exploration of the interplay between reinforcement learning and working memory.

Working memory, vital for flexible behaviors, involves the temporary maintenance and manipulation of information. The prefrontal cortex plays a key role in WM processes, with changes in PFC neural activity correlating with deficits observed in various conditions. While traditional models localized WM to specific brain regions, new perspectives propose a distributed process, with the PFC as a central hub coordinating WM activities. Ongoing research explores how increased delay activity in the PFC contributes to maintaining or controlling information within WM, with reference into processes such as NMDA receptor activation and synaptic dynamics as potential mechanisms (Wong and Wang, 2006; Li et al., 2023). Studies beyond the prefrontal cortex have explored the neural correlates of working memory. Investigations utilizing a simultaneous EEG-fMRI approach have examined the role of the thalamus during the WM delay period, providing insights into subcortical involvement in WM processes. These studies emphasize the importance of considering the broader neural network in understanding WM dynamics (Gomes et al., 2023). The concept of memory retrieval is studied in the context of guiding present and future behaviors. The value of a memory is dependent on its scanning limited segments of stored information and applying them. Terms such as level of awareness, internal attention, working memory, and transient buffer are introduced to characterize this process. An exploration of memory, focusing on hippocampal physiology and its role in supporting various memory operations, is conducted in Buzsáki et al. (2022).

The nature of WM and its interaction with or contribution to RL has been viewed somehow differently in some studies in which the critical role of dopamine in WM is reviewed. In a model called prefrontal basal ganglia or PBWM model, the DA signaling dynamically helps PFC in manipulating the relevant maintained information through a gating process in a somewhat complicated learning task such as 1-2-AX which is a working memory task as well (O’Reilly and Frank, 2006; O’Reilly et al., 2007). So, it seems the whole path is responsible for WM despite that striatum was attributed to RL process. Also in a new study (Li et al., 2022), WM is related to white matter in internal capsule and therefore to structural connections in basal ganglia.

The relationship between reinforcement learning and working memory is shown to be evident in the exploration of arbitrary visuomotor learning, using a dual-system computational model, a habitual system with a Q-Learning algorithm and a goal-directed system employed by a Bayesian Working Memory (BWM) model (Viejo et al., 2015). The model, grounded in Bayesian formalism and Shannon entropy, measures uncertainty in working memory processes. An arbitration process controls the dynamics between Q-Learning and BWM, suggesting an interactive mechanism where memory manipulation is related to the habitual learning. Behavioral results from an instrumental learning task indicates the need for model combinations to thoroughly explain human behavior.

The mechanism involved in associative learning, be it working memory or interaction of reinforcement learning and working memory has been modeled in various fields (Yoo and Collins, 2021) from behavior-focused cognitive sciences to neuroscience focusing on brain networks and to AI and neural networks viewpoints (Baddeley, 1992; Brunel and Wang, 2001; Ashby et al., 2005; Gluck and Pew, 2005; Dash et al., 2007; Cowan, 2012; Bouchacourt and Buschman, 2019; McDougle and Collins, 2020; Kruijne et al., 2021). Some of them are applied in machine learning and some are used in psychiatry and pharmacology to model and predict the effects of drugs or deficiencies on the brain and hence on the behavior. An important source of cognitive impairment that affects learning and working memory is age (Blasiman and Was, 2018). The effect of age on cognitive functions (Van De Vijver et al., 2015), specifically the ones related to frontostriatal brain networks (Eppinger et al., 2013; van de Vijver et al., 2016) and WM related brain networks in the prefrontal cortex (Barbey et al., 2013; Braver and West, 2015; Funahashi, 2017) has been the focus of several studies. The change in the contribution of RL and WM with learning timescale in older adults was explored in van de Vijver and Ligneul (2019).

In Edde et al. (2021), the middle-age around age 40 is considered significant as it involves restructuring of connections between different brain networks that are widespread and eventually contribute to cognitive impairment as individuals age. Most researchers investigated the comparison between younger adults below 30 and older adults above 60, while there is a limited number of studies that focus on contrasting young and middle-aged individuals (Dyussenbayev, 2017). One of them (Siman-Tov et al., 2017) found differences in network interactions between young (21–40) and middle-aged adults (41–61). Similarly, Varangis et al. (2019) investigated brain network dynamics across age in 4 groups including young adults (20–34), younger middle-aged adults (35–49), older middle-aged adults (50–64) and older adults (65+). The study Cao et al. (2014) shows changes in the overall pattern in brain connectivity across almost the whole human lifespan. They observed U-shaped trend in brain connections, with peaks around the age of 40 and 45. Also, the “old age” is recommended as above 65 years by the International Organization for Standardization (ISO) (ISO Standardization Foresight Framework Trend Report, 2022).

By drawing insights from previous studies, we arrived at an age categorization as: 18 to 40 as young adults, 41 to 65 as middle-aged adults, and 65 and above as older adults. This approach enables us to thoroughly explore the influence of age on the phenomena under examination.

This cognitive decline might occur even in healthy aging. A tool that is used to assess cognitive abilities is Montreal Cognitive Assessment test (Nasreddine et al., 2005). The score range from 0 to 30 and normal score is 26 and above. Scores between 18 and 26 are considered Mild Cognitive Impairment and below them could be Alzheimer’s. Since MCI is a stage between normal aging and dementia, the investigation of cognitive state could be helpful since it may lead to dementia. In this study, we seek to find out the changing contribution of RL and WM in older adult with normal cognitive score and MCI.

The first goal of this study was to investigate whether the performance and speed of learning differs significantly in young and middle-aged groups overall and if it is consistent with some previous studies (Salthouse, 1994; van de Vijver and Ligneul, 2019). Next question that we wanted to address was if the middle-aged people in different category of normal and MCI based on MoCA test, differed significantly in performance and speed of learning or the reaction time. Finally, RLWM computational model was fitted to subjects’ performance data in order to further dig into the model parameters which were separately indicative of RL and WM subsystem of associative learning. We will do all these analyzes across age as well to see whether in the whole life span or in specific group, the aforementioned variables have relation with age.

Additionally, we explored the connection between reaction time and the speed of learning and the effort invested in making choices in order to get a comprehensive understanding of the interplay between cognitive systems and learning dynamics.

## 2 Materials and methods

### 2.1 Participants

Subjects were selected from residents in Ashtian province, Iran and Tehran with a total of sixty people aged 25 to 65 participating in this study. Twelve people were excluded from the analysis because they either were unable to complete the task or did not learn and apply the rules of the task. To determine participants’ performance, the examiner used a set of criteria that included evaluating accuracy, response times, and other relevant metrics. Specifically, responses below 33% were considered indicative of a random or chance-level learning process. This threshold was chosen to distinguish participants who demonstrated a genuine effort to learn and apply the rules of the task from those whose responses suggested a lack of engagement or understanding. All subjects were free of neuropsychological problems and did not take psychotherapeutic drugs and had no history of head trauma. They were also given a gift card at the end to compensate for participating in the experiment.

All participants had informed consent to perform a standard computerized task (RLWM task) and a test on paper (MoCA test). The procedure was approved by Research Ethics Committees of Allameh Tabatabaei University with the Approval ID IR.ATU.REC.1400.059. Our study consists a total of 48 individuals, with an age range of 25–65 years (mean 45 ± 12.78 years). The participants were categorized into two groups based on age: the young group, consisting of 22 individuals, and the middle-aged group, comprising 26 individuals. Within the middle-aged group, 11 individuals were healthy, and 15 individuals were identified as having MCI.

In order to categorize age groups, as well as looking into literature mentioned above, we also used logistic regression model to predict the age group based on performance metrics, including reaction time and performance for different sizes. The logistic regression model, formulated as Logit [P(Aged)] = β0 + βRT2 × RT2 + βRT3 × RT3 + βRT4 × RT4 + βRT5 × RT5 + βCorrect2 × Correct2 + βCorrect3 × Correct3 + βCorrect4 × Correct4 + βCorrect5 × Correct5, was trained and tested using the train_test_split method. Applying cut-off values from 35 to 44 to create the ‘aged’ target variable, we found the highest accuracy (85.7%) at an age cut-off of 40, aligning with established milestones for middle age in the literature. Here, RT2, RT3, RT4, and RT5 represent reaction times for different set sizes, and Correct2, Correct3, Correct4, and Correct5 represent accuracy for those respective set sizes. To address the concern about potential double-dipping, the logistic regression analysis was conducted independently of subsequent analyzes examining age-performance metric relationships. Distinct datasets were employed for logistic regression. This reinforces the robustness of our approach.

The logistic regression model predicted age categories with an “aged” label indicating whether a subject exceeded the specific cut-off. Interestingly, the highest accuracy (85.7%) was achieved with age cut-off at 40. This is in alignment with previous studies suggesting 40 as a milestone and starting point of middle age. Another categorization considered in the data analysis is derived from subjects’ cognitive scores in the MoCA test. Specifically, the “Young” group consists of individuals with normal MoCA scores. Middle-aged group subjects are categorized in normal and MCI groups. So, in the remainder of this study, we will include young, middle-aged, normal middle-aged and MCI middle-aged grouping for examining and interpreting our findings.

### 2.2 Procedure

**Figure 1:**
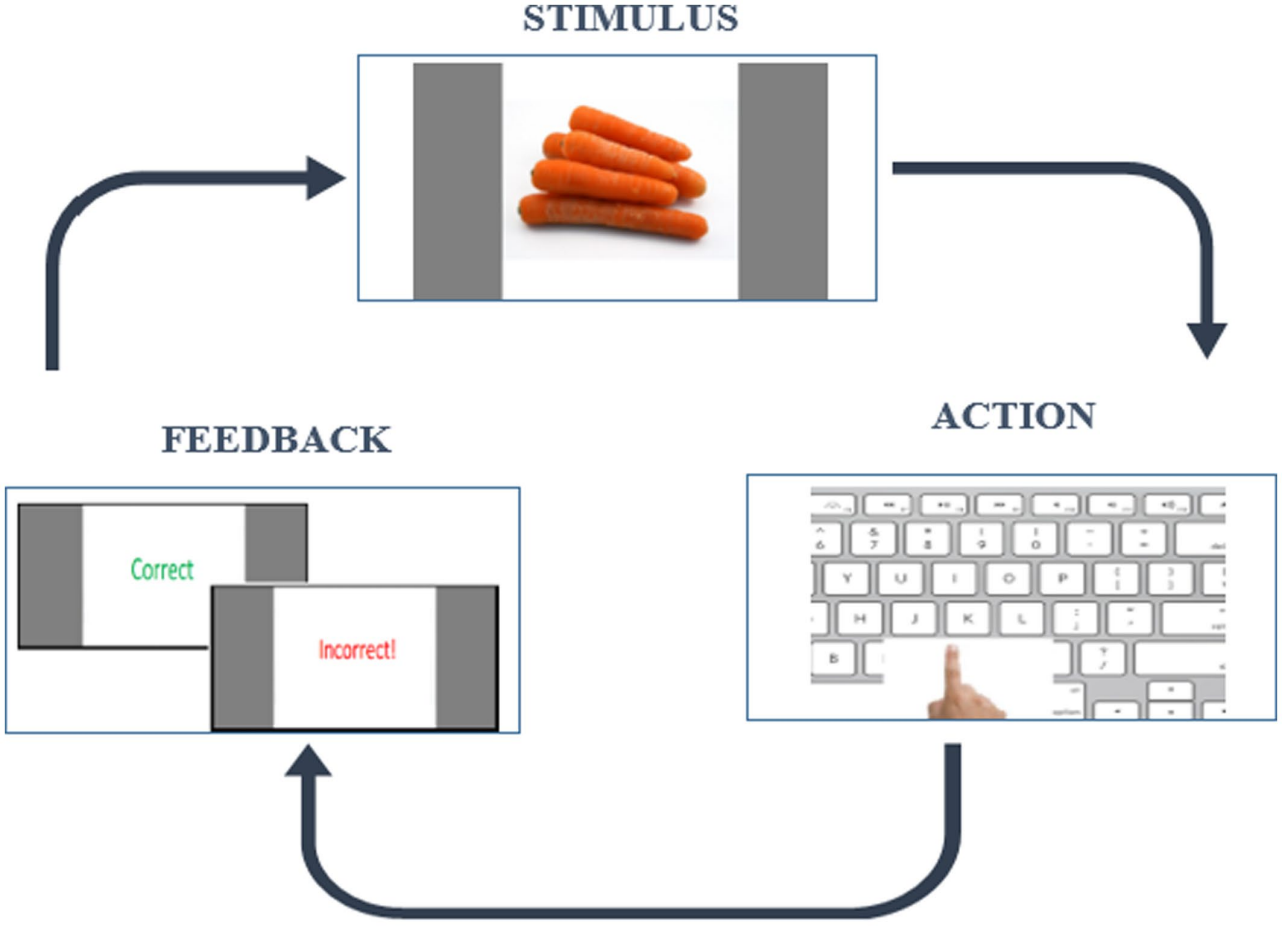
Example of a trial in a block in RLWM task.

We applied the RLWM task which is designed to address the entanglement of RL and WM processes. It is capable of extracting effects of delay and set size on a WM system and hence the related RLWM computational model will formally account for these effects and the dynamic interaction between these two systems. The short version of RLWM task (Master et al., 2020) was selected for this study due to its suitability for a wide age range. It follows an instrumental learning paradigm and originally was designed in the study of Collins and Frank (2012). The task comprised one training block and ten independent learning blocks each containing different categories of visual images, lasting less than 25 min with a total of 468 trials. In each block, subjects encounter a new set of visual stimuli of varying set sizes (ns) from 2 to 5. These stimuli, presented 12–14 times in a pseudo-randomly interleaved manner, are drawn from a single category of familiar and easily identifiable images (e.g., colors, fruits, animals) across 10 different categories. Participants have 7 s to respond by pressing one of three buttons on the keyboard (“J,” “K,” “L”). Feedback, displayed for 0.75 s, includes “Correct” for the accurate key press and “Incorrect!” for an incorrect key press, followed by a fixation period of 0.5 s before the next trial. Failure to answer within 7 s was indicated by a “No valid answer” message. An example trial is shown in Figure 1. The task was implemented in the Psychopy environment (Peirce and MacAskill, 2018), a software package used by neuroscience researchers for designing behavioral experiments.

## 3 Computational modeling

The RLWM computational model first introduced by Collins and Frank (2012) and used in later studies (Collins et al., 2014, 2017; Collins, 2017; Gold et al., 2017; Collins and Frank, 2018; Haile et al., 2020; McDougle and Collins, 2020; Master et al., 2020), formalizes the interaction between two RL and WM systems and put the contribution of each in one model. It assumes each RL and WM has a probability to take over the whole system which shown by P_WM_ and P_RL_. Also, each of them has a policy of choosing an action given their values (Q-values) and is shown by π_WM_ and π_RL_ (Gold et al., 2017). Hence, the overall policy is given in Equation 1:


(1) $$\pi =P_{WM}*\pi_{WM}+\left(1-P_{WM}\right)*\pi_{RL}$$

Assuming that WM has a limited capacity K, it can hold up to K stimulus. So, the probability of WM be chosen is given in Equation 2:


(2) $$P_{WM}=\rho *\min\left(1.\frac{K}{n_{S}}\right)$$

Where ρ is prior parameter. The higher its value, the more contribution WM has in the first place.

Both policies are softmax functions with parameter *β* as the inverse temperature function as given in Equation 3.


(3) $$\pi =\frac{\exp\left(\beta Q\left(s.a\right)\right)}{\sum_{i}\exp\left(\beta Q\left(s.a_{i}\right)\right)}$$

Q-values in RL system will update according to delta rule (McDougle and Collins, 2020) which are given in Equations 4, 5:


(4) $$Q\left(s_{t}.a_{t}\right)\leftarrow Q\left(s_{t}.a_{t}\right)+\alpha_{RL}\times \delta_{t}$$

(5) $$\delta_{t}=r_{t}-Q\left(s_{t}.a_{t}\right)$$

In WM, the Q-values have a learning rate equal to 1 since the learning in WM is immediate. The point is they will face decay and so will update according to Equation 6:


(6) $$Q_{WM}\leftarrow Q_{WM}+\varphi *\left(Q_{0}-Q_{WM}\right)$$

Parameter φ is the decay or forgetting parameter in WM system. Initial value Q_0_ is equal to 1/n_A_, n_A_ is the number of possible actions and equals 3(j, k, l). There is also a noise parameter ε called undirected noise and affects the policy and is given in Equation 7:


(7) $$\pi^{\prime }=\left(1-\varepsilon \right)\;\pi +\varepsilon U$$

and U = 1/n_A_, the uniform random policy.

This model employs 6 free parameters for characterizing behavior from which 4 are considered in this study and include RL learning rate (α), WM decay (φ), WM prior weight and undirected noise (ε). The last one, parameter “epsilon” is chosen to capture decision noise which is independent of learning process.

## 4 Results

### 4.1 Behavioral analysis

**Figure 2:**
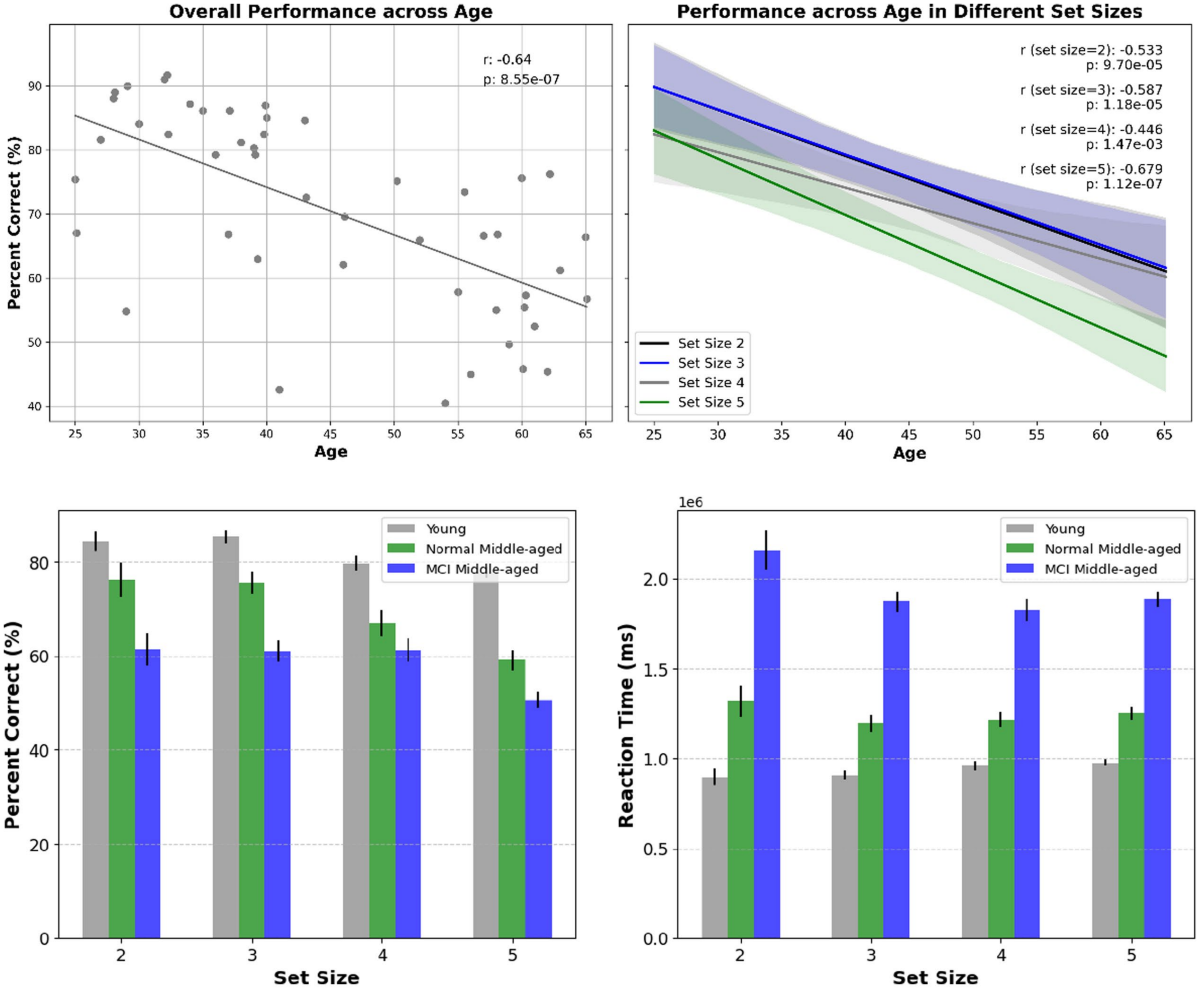
Top panel: overall performance across age and the correlation within subjects (left) and for different set sizes across age (right). Statistical significance shows that age has a substantial effect on overall performance as well as performance in various set sizes. Bottom panel: overall performance for different set sizes and across different cognitive status (left) reaction time for different set sizes and across different cognitive status (right).

The first behavior is related to the overall correct answers or the performance accuracy denoted as percent correct. In Figure 2 it is shown for all set sizes across participants of different ages and for different groups. The overall performance decreases significantly with increasing age and set size in all groups. We employed the linear regression analysis and it is revealed that there is a significant correlation among all subjects’ performance as they age (r-squared = 0.41, *p*-value < 0.01). Also, analyzing performance across age for all young and normal middle-aged (excluding MCI subjects) gives a correlation coefficient r-squared = 0.18 with *p*-value < 0.01. However, among middle-aged participants no significant correlation across age observed (r-squared = 0.05, *p*-value = 0.3). This lack of age-related correlation was true among young subjects (r-squared = 0.002, *p*-value = 0.84), normal middle-aged subjects (r-squared = 0, *p*-value = 0.98) and normal middle-aged subjects (r-squared = 0, *p*-value = 0.96).

To assess performance across groups, first the normality assumptions were examined by Shapiro–Wilk test. It was revealed that young group data deviated from normality (*p*-value = 0.004) while middle-aged group showed no deviation (*p*-value = 0.613). Subsequently, non-parametric Mann–Whitney U test was used for comparison and the results showed a statistically significant difference between young and normal middle-aged groups (*p*-value = 0.00196) as well as young and all middle-aged group. Furthermore, contrasting performance between normal and MCI middle-aged group, showed a significant difference too (*p*-value = 0.0375).

**Figure 3:**
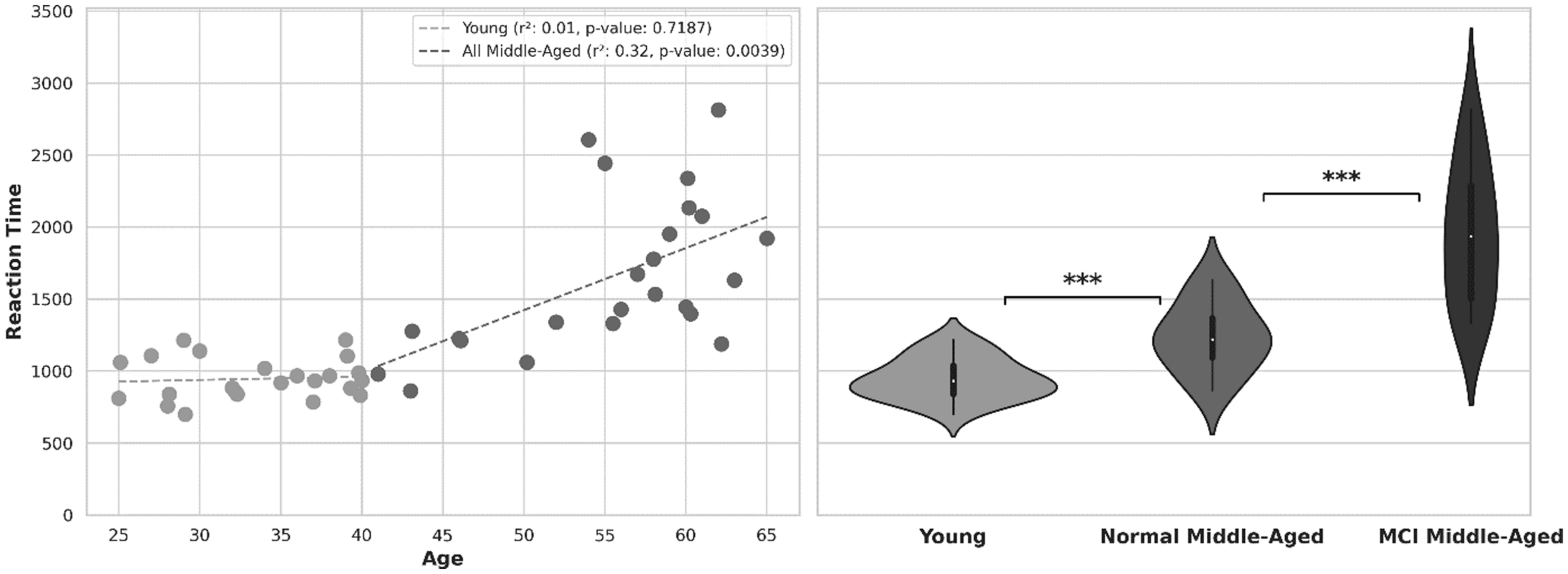
Left: reaction time across age. Right: violin plot showing the distribution of reaction time in 3 groups.

Another behavioral indicator, reaction time or the time taken to respond to stimuli is depicted in Figure 3. The scatter plot shows within subject’s correlation between reaction time and age, with separate regression lines related to young and middle-aged groups. For all participants, there was a noteworthy increase in reaction time as their age increased (r-squared = 0.54 with *p*-value < 0.01). this holds true among all normal (young and middle aged excluding MCI) participants (r-squared = 0.48 with *p*-value < 0.01). For young group, there is no significant correlation (*p*-value = 0.72) and within subjects’ correlation is weak. Within all subjects in the middle-aged group, there is a moderate degree of correlation (r-squared = 0.32, *p*-value = 0.0039) as they age. Analyzing reaction time across age for normal middle-aged (excluding MCI subjects) gives a correlation coefficient r-squared = 0.51 with *p*-value = 0.0198. However, among MCI middle-aged participants, no significant correlation across age observed (r-squared = 0.08, *p*-value = 0.32).

The violin plot in Figure 3 provides distribution information and after normality test, Welch’s t-test shows the statistical significance of disparity in reaction time between young and all middle-aged subjects (value of *p*<0.01). After excluding MCI scores out of middle-age subjects, there is still a significant difference between young group and normal middle-aged group (*p*-value = 0.0037). Subsequently, comparing reaction times between middle-aged subjects with MCI and normal ones, there was a significant distinction (*p*-value = 0.00016). In the overall analysis across all ages, the calculated power was 0.79. However, when analyzing middle-aged group separately, the estimated power was approximately 0.45.

**Figure 4:**
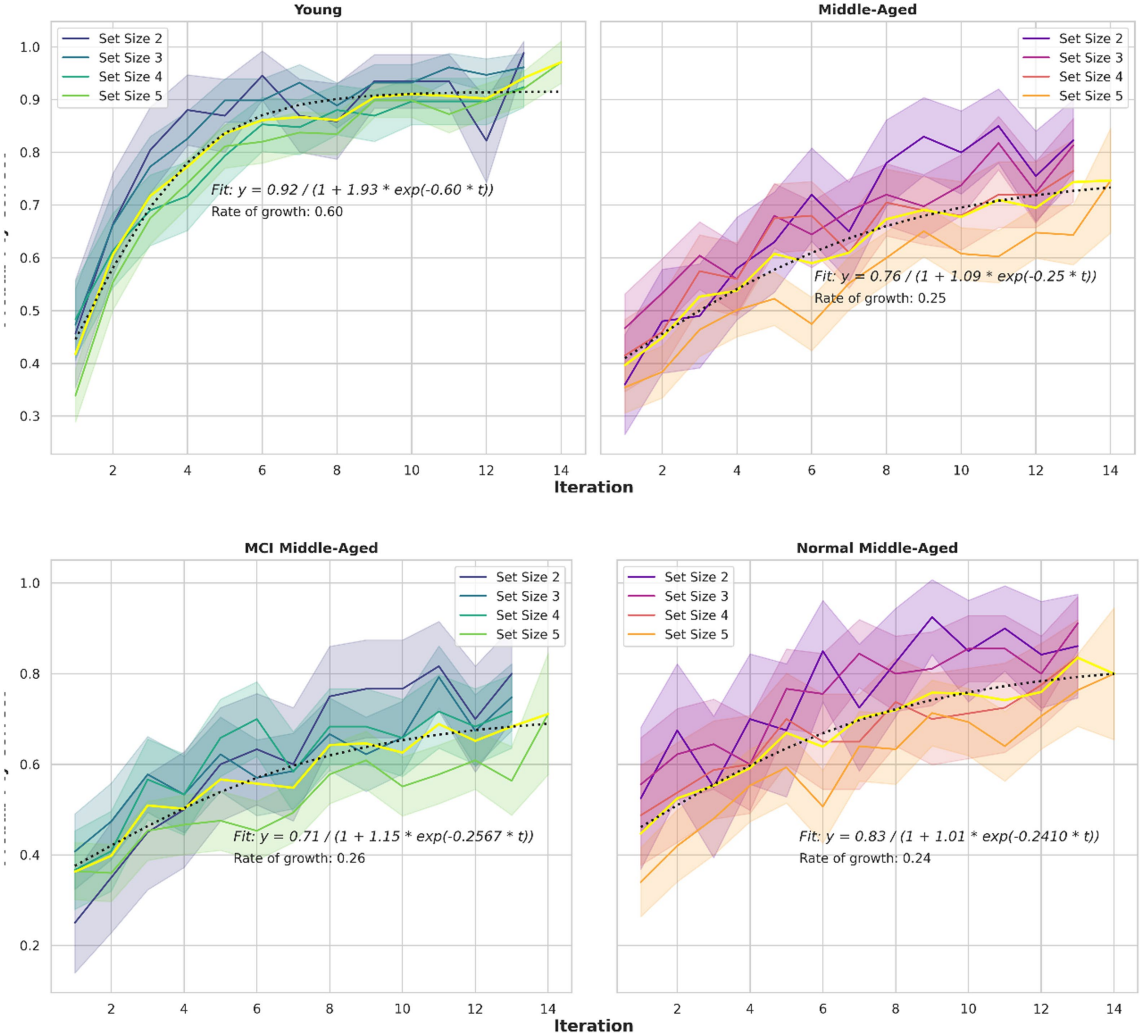
Learning curves for different set sizes and two groups. The logistic growth model fitted to learning curve data for all set sizes in various groups, depicted in yellow. The equations and related parameters are displayed. Top panel compares young group and all middle-aged subjects including normal and MCI. Middle panel compares young and normal middle-aged. Their ultimate performance differs slightly but the speed to reach that performance is nearly 3 times larger for the young adults (0.6048 vs. 0.241). Bottom panel compare normal and MCI within middle-aged group. Their speed is similar but the final performance differs (0.83 vs. 0.71).

A third behavioral metric is the learning curve, as illustrated in Figure 4. Learning curves depict the correct percentage of trials as a function of trials or iterations completed. Consistent with previous studies (Collins and Frank, 2012; van de Vijver and Ligneul, 2019; Master et al., 2020), participants reached asymptotic performance more rapidly in small set sizes than in larger ones. This pattern is evident across all groups with a slight deviation for the middle-aged group. Also, the young group, showed faster learning, indicating a difference in the speed with which the learning curve reaches its asymptote, or the curve’s time constant. To assess these observations quantitatively, a mathematical model is applied and key parameters are extracted. Specifically, a logistic growth model described by the equation P(t) = A/(1 + B*exp. (−k*t)) is assumed. The parameters are A(asymptote), B (scaling) and k (growth rate or time constant). This growth model is fitted to the learning curves.

For model fitting, the RLWM model was fit to the subjects’ data using maximum likelihood method with Python scipy.optimize package. The correlation matrix of parameters in different groups is given in Table 1. It is evident that no strong significant correlation exists between parameters.

**Table 1:** Parameters’ correlation matrix.

		Alpha	Prior	Epsilon	Phi
Young group	Alpha	1	0.03^*^	0.77	0.01^**^
	Prior		1	0.013	0.01^*^
	Epsilon			1	0.03^*^
	Phi				1
Normal middle-aged group	Alpha	1	0.01^*^	0.01^*^	0.01^*^
	Prior		1	0.02^*^	0.23
	Epsilon			1	0.02^*^
	Phi				1
MCI middle-aged group	Alpha	1	0.04^*^	0.03^*^	0.56
	Prior		1	0.09	0.36
	Epsilon			1	0.02^*^
	Phi				1

### 4.2 The effect of age and MoCA status on model parameters

**Figure 5:**
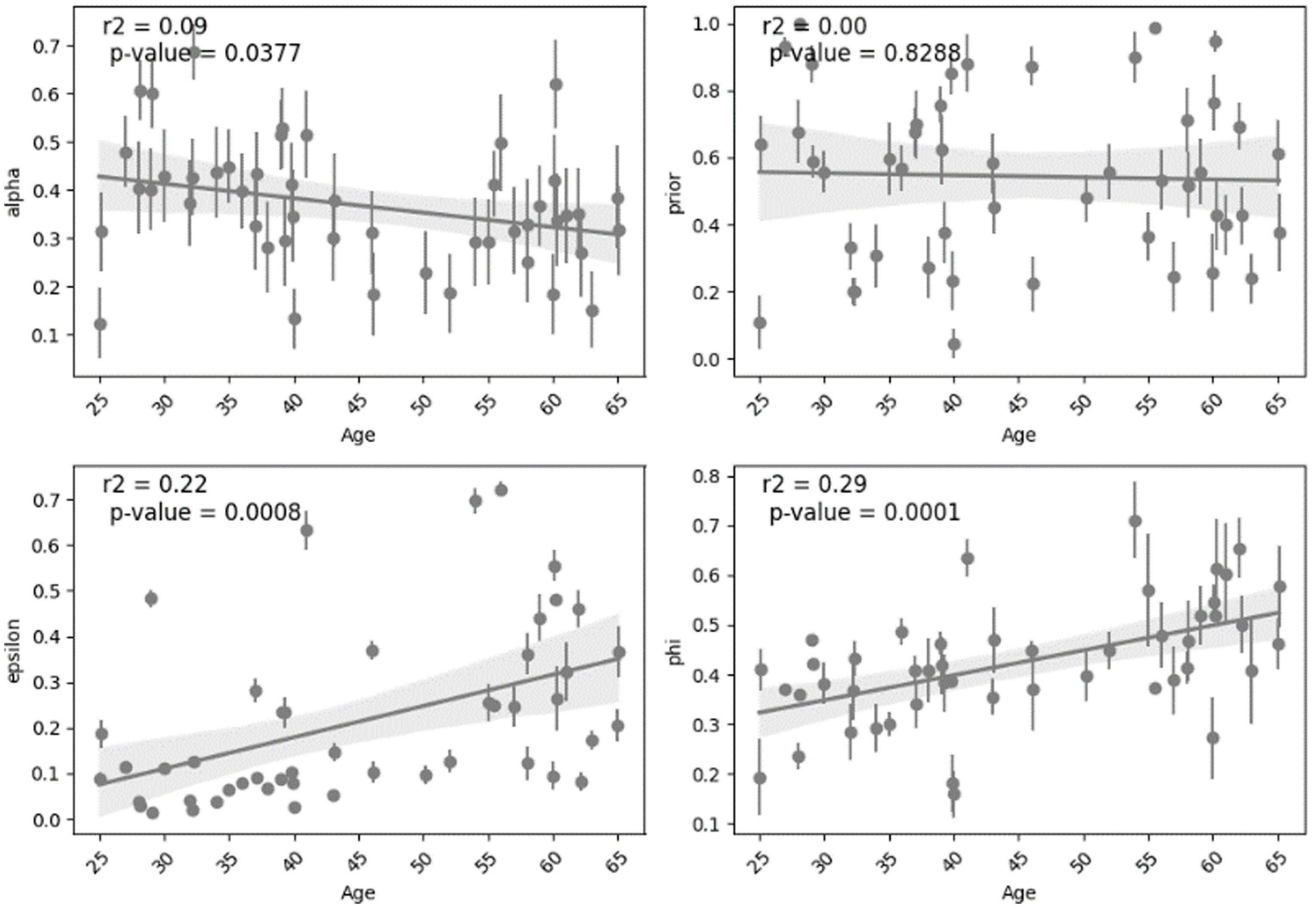
The trends observed in the fitted parameters with respect to age across all subjects.

Analyzing the relationship between age and the parameters shows different effect sizes. The parameters “alpha” and “prior” show weak effect sizes with lower Spearman’s coefficients (0.17 and 0.05) and small r-squared (0.09 and 0.001). The parameters “epsilon” and “phi” exhibit stronger effect sizes indicated by Spearman’s coefficients (0.39 and 0.34) and larger r-squared (0.22 and 0.29) which can suggest more highlighted relationship with age. This analysis was done across all subjects and is shown in Figure 5. For other cases such as all normal subjects (MCI excluded), all young, all middle-aged, normal middle-aged and MCI middle-aged, this correlation and regression analysis is implemented and summarized in Table 2.

**Table 2:** Parameters’ correlation/regression analysis across age.

Subjects		Alpha	Prior	Epsilon	Phi
Young and normal Middle-aged	Spearman *ρ* (*p*-value)	−0.22 (<0.001)	−0.15 (<0.001)	0.19 (<0.001)	0.24 (<0.001)
	r-squared (*p*-value)	0.21 (0.008)	0.042(0.26)	0.02 (0.43)	0.175 (0.015)
Young	Spearman *ρ* (*p*-value)	−0.05 (0.13)	−0.18 (<0.001)	0.1 (0.002)	0.014 (0.66)
	r-squared (*p*-value)	0.01 (0.6)	0.04(0.36)	0 (0.98)	0 (0.98)
All middle-aged	Spearman *ρ* (*p*-value)	−0.02 (0.6)	−0.15 (<0.001)	0.03 (0.32)	0.11 (<0.001)
	r-squared (*p*-value)	0 (0.95)	0.04 (0.37)	0 (0.76)	0.03 (0.4)
Normal middle-aged	Spearman *ρ* (*p*-value)	−0.22 (<0.001)	−0.4 (<0.001)	−0.24 (<0.001)	−0.03 (0.6)
	r-squared (*p*-value)	0.28 (0.12)	0.27 (0.12)	0.11 (0.35)	0.01 (0.76)
MCI middle-aged	Spearman *ρ* (*p*-valuep)	−0.06 (0.14)	−0.09 (0.04)	0.006 (0.9)	0.15 (<0.001)
	r-squared (*p*-value)	0.05 (0.4)	0.02 (0.6)	0 (0.8)	0.01 (0.7)

Overall, we see that in some cases the correlation between a parameter and age is statistically significant based on Spearman value of p but the linear regression model’s ability to predict that parameter from age might not be statistically significant. In case of all normal subjects (excluding MCI), there is a robust and significant decline in “alpha” and growth in “phi” with age for both correlation and regression analysis. In other situations, age is not a significant predictor of parameters regarding linear regression model but is correlated in some cases as shown in Table 2.

In examining the relationship between age and epsilon, a significant difference emerged between the young and middle-aged groups (t-statistic = −3.64, value of *p* = 6.9e-04). After applying the Bonferroni correction to account for multiple comparisons, the corrected value of p remained remarkably low (6.9e-04). In relating between age and phi using linear regression, a significant difference existed between the young and middle-aged groups (t-statistic = −3.61, value of *p* = 7.5e-04). After applying the Bonferroni correction to account for multiple comparisons, the corrected *p*-value remained remarkably low (7.5e-04). This indicates a robust statistical significance.

**Figure 6:**
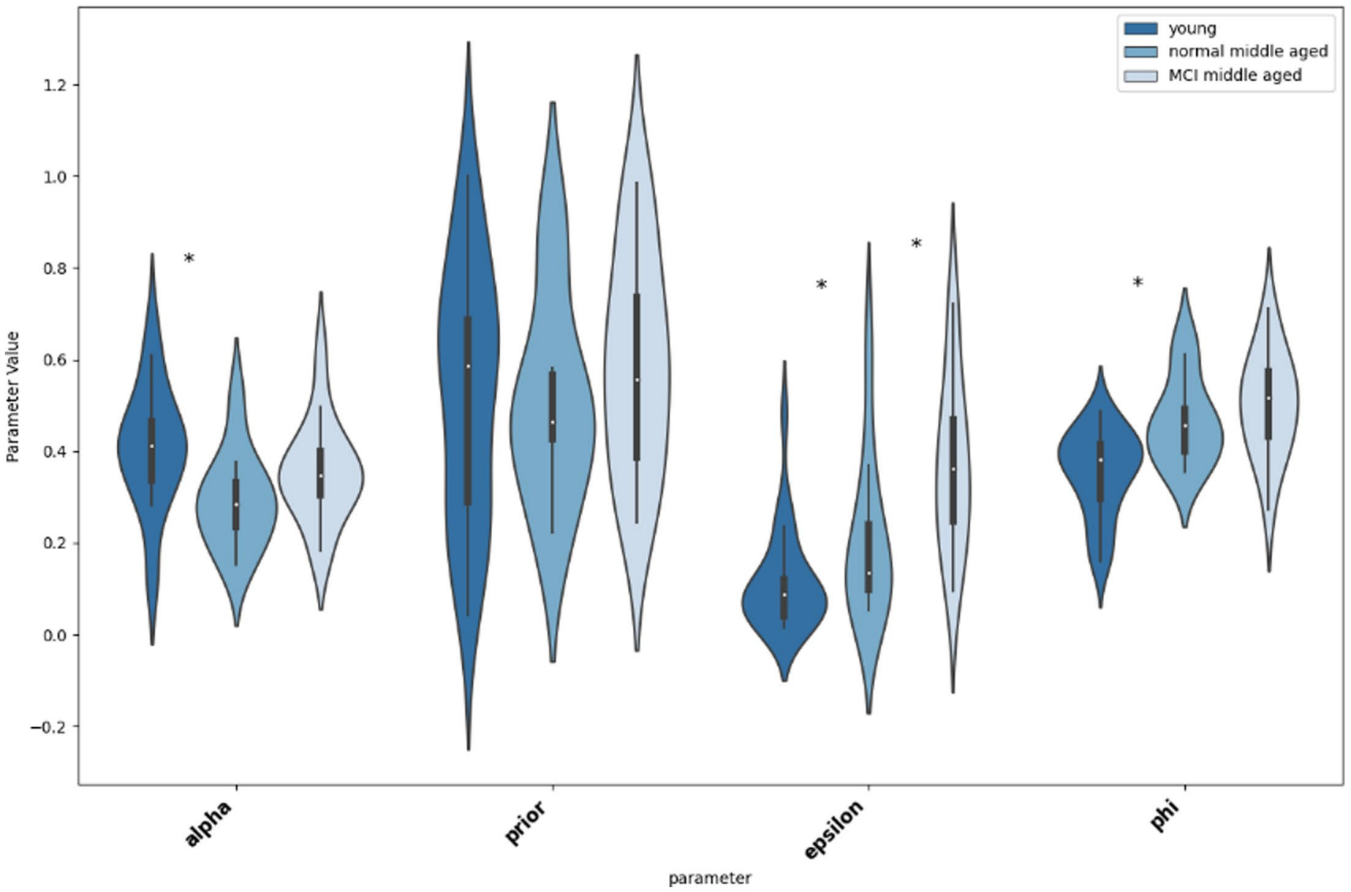
Violin plot showing the distribution and significance of difference in parameter values between groups. The asterisk indicates statistical significance.

Thereafter, we compared the parameters for different groups, first young and normal middle-aged, as their difference is due to their age group, and then normal and MCI middle-aged group that their difference is their MoCA score. As shown in Figure 6, according to the Mann–Whitney U tests, there is significant difference between young and normal middle-aged group in parameters “alpha” (*p* = 0.0129, Cohen’s d = 0.56), “epsilon” (*p* = 0.0479, Cohen’s d = −0.44) and “phi” (*p* = 0.016, Cohen’s d = −0.54). A practical significance exists too as indicated by moderate effect size. On the other hand, no statistical significance as well as small effect size (*p* = 0.74, Cohen’s d = 0.08) for parameter ‘prior’ between young and normal middle-aged group is observed. Then, considering differences between normal and MCI groups in middle-aged subjects, the only statistical significant difference is in parameter “epsilon” (*p* = 0.028, Cohen’s d = −0.53). Other parameters do not differ between normal and MCI groups (‘alpha’: *p* = 0.11, Cohen’s d = −0.4, “prior: *p* = 0.33, Cohen’s d = −0.24, ‘phi: *p* = 0.33, Cohen’s d = −0.24).

### 4.3 The relationship between learning curves and model parameters

As the observation revealed in the previous part, alpha and phi exhibit distinctions between young and normal middle-aged group, yet remain consistent across normal and MCI middle-aged groups. This pattern is similar to the analysis of learning curves among the aforementioned groups. Interestingly, learning curves’ time constant parameter k (the learning curve’s time constant or rate of growth shown in Figure 4) is comparable among normal and MCI middle-aged groups, while notably greater in the young group compared to normal middle-aged group. Since these two parameters (alpha as RL learning rate and phi as WM decay rate) are rate-based factors, they can be linked to other variables governed by speed or rate-based metrics, such as time constant or reaction time. Notably, the ratio of mean alpha for young per normal groups divided by mean phi of young per normal group is equal to the product of tau ratio of young per normal middle-aged and the RT ratio. In other form, we have:


$$\frac{\frac{\alpha_{\mathrm{young}}}{\alpha_{\mathrm{normal}\;\mathrm{middle}-\mathrm{aged}}}}{\frac{\varphi_{\mathrm{young}}}{\varphi_{\mathrm{normal}\;\mathrm{middle}-\mathrm{aged}}}}=\frac{\tau_{\mathrm{young}}}{\tau_{\mathrm{normal}\;\mathrm{middle}-\mathrm{aged}}}*\frac{RT_{\mathrm{young}}}{RT_{\mathrm{normal}\;\mathrm{middle}-\mathrm{aged}}}$$

This equivalence suggests that the balance between learning and memory processes is proportional to the combined relevance of learning speed differences and reaction speed differences.

Utilizing an analysis of variance (ANOVA) approach, we examined the growth rate parameter (k) and the asymptote parameter (A) obtained from the logistic growth model fitted to individual learning curves. The results revealed statistically significant differences between the two age groups for both k (F-statistic = 11.65, *p*-value = 0.00135) and A (F-statistic = 16.89, *p*-value = 0.00016). *Post hoc* Tukey Honestly Significant Difference (HSD) tests were conducted to further explore pairwise group differences. For the growth rate parameter (k), the mean difference between the middle-aged and young groups was 0.1377 (*p*-adj = 0.0014), and for the asymptote parameter (A), the mean difference was 0.1536 (*p*-adj = 0.0002). These outcomes show the statistical significance of group differences and enhance the robustness of our findings.

### 4.4 Speed vs. accuracy

It is shown that in reaction time analysis, incorrect responses are faster than correct ones. In a study (Statsenko et al., 2020), another trend was observed for individuals under 20 and above 60, where longer reaction times were associated with more errors, while the traditional speed-accuracy was held true for subjects between ages of 20 and 60. In this study, when we focused on young and normal middle-aged group, no speed-accuracy tradeoff was observed. However, among MCI group, with increasing reaction time, performance increased (r-squared = 0.36, *p* = 0.02). Also, the mean RT for correct and error responses were computed for each group and in all of the error responses took longer than correct responses on average. Even after a specific iteration where the learning curve’s logistic growth model with group-specific parameters reached a threshold of 10% it’s max value (which can be called the initiation of learning and was iteration 4 for young group and iteration 9 for normal and MCI middle-aged groups), the error RT was still more than correct RT in all young, normal middle-aged and MCI middle-aged groups on average.

Then, we computed the inverse efficiency score (IES) measure for the groups under study. It combines speed and accuracy in tasks by dividing reaction time to percent correct responses (Bruyer and Brysbaert, 2011). Here, there was no relationship between age and IES in neither young, normal and MCI groups. On the other hand, there was a significant difference in IES between young and normal group (Mann–Whitney *p* < 0.01) and normal and MCI group (Mann–Whitney *p* < 0.01).

## 5 Discussion

Within the broad field of aging research, our study contributes to the understanding of the dynamic interplay between Learning and Working Memory systems across a range of age groups. We investigate age-related changes in RLWM parameters using computational modeling. The foundational insights from years of research on cognitive aging (Anderson and Craik, 2017; Ebaid and Crewther, 2020; McDonough et al., 2022) provide a comprehensive framework, incorporating factors such as processing speed and compensatory mechanisms. Notable declines in various cognitive domains such as working memory capacity, processing speed, learning and reasoning by aging are revealed in multiple modeling studies (Li and Sikström, 2002; Goh and Park, 2009). Our observed changes in RLWM parameters, phi and alpha, agree with the notion of processing speed (Salthouse, 1996; Eckert, 2011; Albinet et al., 2012). According to this theoretical paradigm, changes in processing speed are closely related to age-related changes in cognitive function and is also aligned with an observational study that showed aging-related decreases in working memory and processing speed (Park et al., 2002).

Another recent study (van de Vijver and Ligneul, 2019) that explored the effects of aging on cognitive systems, including updating and maintaining stimulus–response associations, found that age has no direct impact on decision-release parameters. The stability of the inverse temperature controlling value-driven choice and random noise controlling value-independent exploration across age groups emphasizes the resilience of certain decision-making components in both young and older adults. Our analysis shows that RL and WM parameters (alpha, phi, and decision noise) change with age. We find higher WM decay rates in higher ages that suggests a deterioration in the maintenance of stimulus–response association. In line with some studies, the use of prefrontal executive system is less frequent as people age. In addition, our research takes into account the effect of lower level processes such as dopamine signaling, known to control learning rates. With aging, a dysregulation of dopamine receptors in the frontal regions is proposed to cause frontal neural noise (Kaasinen et al., 2000; Goh and Park, 2009; van de Vijver and Ligneul, 2019). In the context of attention and choice, it is demonstrated that older adults have difficulty in ignoring irrelevant information during target selection (Rabbitt, 1965). Also, it is proposed that older adults are less able to inhibit unwanted material, which may occupy working memory space, reducing temporary storage and further processing abilities (Hasher and Zacks, 1988). The nature of inhibition is acknowledged as multifaceted and can be related to decision noise.

The primary objective of this research was to examine how reinforcement learning (RL) and working memory (WM) systems interact across the lifespan, focusing on the often overlooked middle-aged subjects. While previous studies have explored this interaction in subjects aged 8–17 and 25–30 (Master et al., 2020) or for young individuals aged 18–35 and older adults above 65 (van de Vijver and Ligneul, 2019), a gap existed for the middle-aged individuals. So, we aimed to bridge this gap. Additionally, we extended our investigation to include subjects with cognitive impairment.

Since all individuals in the young group have normal MoCA status, a one-way ANOVA is conducted. In the analysis of two dependent variables, response time and performance, the impact of categorical independent variables, age-group and MOCA status, was investigated. Significant findings include the influence of age-group on RT (F-statistic = 5.21, *p* < 0.05), indicating differences in response times among age groups. Similarly, MOCA status significantly affected RT (F-statistic = 31.82, *p* < 0.05). Regarding performance, age-group (F-statistic = 9.39, *p* < 0.05) and MOCA status (F-statistic = 7.55, *p* < 0.05) both exhibited significant effects, highlighting performance variations across age groups and the influence of cognitive status. However, after implementing Bonferroni correction to mitigate the risk of Type I errors associated with multiple comparisons, the significance levels were adjusted to 0.0125. The findings revealed that the influence of age group on both response time and performance remained statistically significant at the adjusted significance level, emphasizing consistent differences across age groups. Conversely, while the initial analysis indicated a significant impact of MOCA status on both response time and performance, these effects were no longer deemed statistically significant after Bonferroni correction. In the analysis of response time, Tukey’s Honestly Significant Difference (HSD) test revealed a significant difference between the middle-aged and young groups (mean difference = −666.57, *p* < 0.05). This result suggests that, on average, the young group exhibits lower response times compared to the middle-aged group, indicating a statistically significant variation in response time between these age groups. Similarly, in the assessment of performance, a significant difference emerged between the middle-aged and young groups (mean difference = 0.1887, *p* < 0.05). The positive mean difference signifies that, on average, the young group demonstrates higher performance compared to the middle-aged group.

Our behavioral results from the regression analysis indicate that there is no significant change in accuracy or speed across age for individuals in their youth. In other words, there was no notable difference in performance and speed between a 39-year-old and a 25-year-old. This is true for the overall performance among the middle-age group as they age. However, in terms of speed, represented by reaction time, a decline was observed across age for middle-aged individuals. This observed decline in reaction time could be attributed to a reduced reliance on working memory (as indicated by prior parameter trends) and an increase in phi (working memory decay rate). Since the prior parameter trends indicate a decline across the young group as they age, it is likely that the responsible factor for this decline is phi.

To investigate this, we examined how phi and prior predict reaction time in two age groups. We performed regression analyzes for each group. For the young group R-squared value was 0.62, indicating that approximately 62% of the variance in reaction time within this group can be explained by the working memory decay rate and the parameter prior. Positive coefficients for phi (722.22) and prior (137.07) suggest that higher values of these variables are associated with longer reaction times in this group. For the middle-aged group we have R-squared value was 0.45 and coefficients for phi (2355.98) and prior (156.34). It shows that the impact of changes in the working memory decay rate on predicted reaction time appears to be more pronounced in the middle-aged group compared to the young group. Higher coefficients suggest a stronger influence of the phi variable on reaction time in the middle-aged group.

The observed variations in reaction time across age groups in our study are in line with previous research investigating the relationship between working memory and reaction time. Existing literature has consistently reported correlations between working memory capacity and reaction time, its association with fluid intelligence (Unsworth and Engle, 2005; Meiran and Shahar, 2018) and the link between RT distribution parameters and constructs of higher cognition such as working memory, reasoning and psychometric speed (Schmiedek et al., 2007). Moreover, the connection between task switching and working memory capacity has been well-established, particularly when considering combined measures of reaction time and accuracy (Draheim et al., 2016).

Analyzing RLWM model parameters across age groups, can shed light on the interaction between cognitive processes and aging. The observed consistency in WM prior weight in all 3 groups, suggests that initial utilization of working memory is independent of age or cognitive status and may remain stable despite middle aging or early cognitive decline. Notably, epsilon (undirected noise) differs significantly between young and normal middle-aged participants, as well as between normal middle-aged and those with MCI. The increased decision noise in normal middle-aged subjects compared to young group can indicate a decline in the individual’s effort or their decreased degree of confidence (Jerjian et al., 2023) that could yield to a decrease in both speed and accuracy of choice. Furthermore, the variations in alpha and phi as rates related to RL and WM separately and their alignment with learning curves’ specifications, shows a potential connection between cognitive processing and learning efficiency that needs a more mechanistic analysis. Additionally, age can be a determinant of processing speed while cognitive status is not. Overall, comparing learning trajectories in 3 groups show a rapid initial learning in young group, along with more prolonged learning in middle-aged groups. But since the ultimate performance in MCI is lower than normal group, while epsilon is significantly higher in MCI group, a role of decision noise can be suggested.

To investigate whether the variation in epsilon is attributed to poor fit, we compared the model fit between the normal middle-aged group and those with MCI (t-statistic = −1.67, *p*-value = 0.126) and it indicates no significant difference. However, an analysis of the relationship between epsilon and negative log-likelihood across all subjects revealed a robust correlation (*r* = −0.86, *p*-value < 0.01). Specifically, this difference in model fit between young and middle-aged group was significant (t-statistic = 4.66, *p*-value < 0.01). This finding implies an age-related variation in how well the model captures the data, which could be consistent with a shift of, or variability in, choice strategies employed by older adults, or simply that the task did not sufficiently engage this group.

The paper makes several recommendations for further investigation. Research that follows RL and WM parameters over time across age, may identify patterns of development and provide detailed information on how these cognitive processes change over time. Furthermore, the effectiveness of reducing age-related changes may be assessed by adopting cognitive intervention programs that focus on specific cognitive systems. The study’s implications may be further expanded by examining the possible role of genetic factors, taking lifestyle and environmental factors into account, and performing comparative research across cognitive domains. These methods provide a thorough approach to understanding age-related cognitive changes.

## Data availability statement

The raw data supporting the conclusions of this article will be made available by the authors, without undue reservation.

## Ethics statement

The studies involving humans were approved by Dr. Abdollah Motamedi, Committee Director, Allameh Tabatabaei University Dr. Seyed Jalal Dehghani Firoozabadi, Committee Secretory, Allameh Tabatabaei University. The studies were conducted in accordance with the local legislation and institutional requirements. The participants provided their written informed consent to participate in this study.

## Author contributions

ZH: Data curation, Methodology, Software, Visualization, Writing – original draft. FB: Supervision, Validation, Writing – review & editing. FD: Supervision, Writing – review & editing.
